# FHBDSR-Net: automated measurement of diseased spikelet rate of Fusarium Head Blight on wheat spikes

**DOI:** 10.1007/s42994-025-00245-0

**Published:** 2025-09-02

**Authors:** Ze Wu, Haowei Zhao, Zeyu Chen, Yongqiang Suo, Seena Joseph, Xiaohui Yuan, Caixia Lan, Weizhen Liu

**Affiliations:** 1 School of Computer Science and Artificial Intelligence, Wuhan University of Technology, Wuhan, 430070 China; 2 Hubei Hongshan Laboratory, College of Plant Science and Technology, Huazhong Agricultural University, Wuhan, 430070 China; 3 School of Applied Computing, Wales Institute of Science and Arts, UWTSD, Swansea, SA1 8EW UK; 4 Yazhouwan National Laboratory, Sanya, 572025 China; 5 Engineering Research Centre of Chinese Ministry of Education for Edible and Medicinal Fungi, Jilin Agricultural University, Changchun, 130118 China; 6 Sanya Science and Education Innovation Park of Wuhan University of Technology, Sanya, 572025 China

**Keywords:** Smart breeding, Wheat Fusarium Head Blight, Diseased spikelet rate, Object detection

## Abstract

Fusarium Head Blight (FHB), a fungal wheat (*Triticum aestivum*) disease that threatens global food security, requires precise quantification of diseased spikelet rate (DSR) as a phenotypic indicator for resistance breeding. Most techniques for measuring DSR rely on manual spikelet-by-spikelet observation and counting, which is inefficient and destructive. Although deep learning offers great promise for automated DSR measurement, existing intelligent detection algorithms are hampered by the lack of spikelet-level annotated data, insufficient feature representation for diseased spikelets, and weak spatial encoding of densely arranged spikelets. To address these challenges, we constructed a dataset of 620 high-resolution RGB images of wheat spikes with 5,222 spikelet-level annotations to systematically analyze spikelet size distributions to fill small-object detection data gaps in this field. We designed FHBDSR-Net, a light framework for automated DSR measurement centered on diseased spikelet detection, which features (1) multi-scale feature enhancement architecture that dynamically combines lesion textures, morphological features, and lesion-awn contrast through adaptive multi-scale kernels to suppress background noise; (2) the Inner-EfficiCIoU loss function to reduce small-target localization errors in dense contexts; and (3) a scale-aware attention module using dilated convolutions and self-attention to encode multi-scale pathological patterns and spatial distributions to enhance dense spikelet resolution. FHBDSR-Net detected diseased spikelets with an average precision of 93.8% with a lightweight design of 7.2 M parameters. The results were strongly correlated with expert evaluations, with a Pearson correlation coefficient of 0.901. Our method is suitable for deployment on resource-constrained mobile devices, facilitating portable plant phenotyping and smart breeding.

## Introduction

Fusarium Head Blight (FHB), also known as scab, is a historically devastating, insidious disease that is prevalent in semi-humid and humid areas worldwide (Chai et al. 2022). Diseased wheat (*Triticum aestivum*) plants suffer from poor germination (Gilbert and Tekauz 2000), flower abortion (Bai and Shaner 2004), and kernel degradation (Góral et al. 2018), leading to serious yield losses and reduced grain quality (Wang et al. 2020). There have been multiple epidemics of FHB (Ma et al. 2020), leading to significant economic losses, such as over $2.492 billion losses in the United States from 1993 to 2001 (Nganje et al. 2004). This disease produces mycotoxins, most notably deoxynivalenol (DON), posing serious threats to agricultural worker safety and public health (Nguyen et al. 2024; O’Donnell et al. 2004; Su et al. 2019). Breeding resistant cultivars represent a major control strategy, with numerous wheat varieties developed from resistant sources such as Sumai 3 (Brar et al. 2019). However, critical challenges remain, particularly the lack of precise, automated methods for scoring FHB-resistant phenotypes in wheat.

**Fig. 1:**
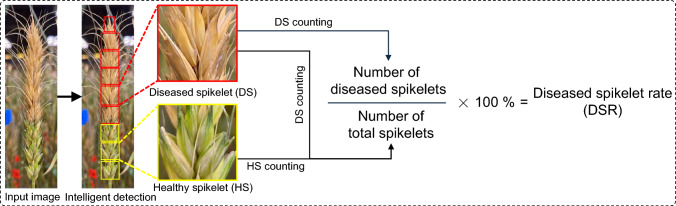
Diagram of the process used to compute diseased spikelet rate (DSR)

The diseased spikelet rate (DSR) is an important spikelet-level phenotype that is widely employed by wheat breeders and plant pathologists to assess the severity of FHB (Mahlein et al. 2019). The DSR, i.e., the proportion of infected spikelets per spike (Fig. 1), is used to monitor disease progression, assess resistance levels, and analyze genotype–trait associations (Miedaner et al. 2024; Powell et al. 2024; Syed et al. 2024). DSR data are currently collected based on manual counting, where researchers must visually inspect each spikelet to determine whether it is infected and then calculate the proportion of diseased spikelets on the entire spike (Wang et al. 2023b). This process is tedious, time-consuming, and highly dependent on visual accuracy and attention, making it prone to error (Buerstmayr et al. 2020; Liu et al. 2019). Thus, developing automated, non-invasive methods for accurate spikelet-level DSR assessment holds significant research value and application prospects.

Thanks to advancements in deep learning, automated approaches for intelligent detection and quantification of FHB in wheat have emerged. By leveraging advanced imaging technologies and intelligent detection algorithms, such as segmentation, classification, and object detection, these methods achieve high precision for disease identification (Wang et al. 2023c; Zhang et al. 2022). Single-stage object detection algorithms, with high accuracy and lightweight design, enable the rapid localization and classification of lesions, offering efficient FHB detection and phenotypic extraction (Bao et al. 2024; Hong et al. 2022; Zhang et al. 2022). An improved YOLOv5 model was proposed to extract wheat spikes from images and subsequently perform disease assessment using threshold segmentation and a random forest binary classifier (Zhang et al. 2022). Hong et al. (2022) designed a lightweight model based on MobileNet and YOLOv4, which can be deployed on unmanned aerial vehicle (UAV) devices to detect FHB in the field. Bao et al. (2024) proposed an enhanced YOLOv5-S lightweight model for identifying and localizing diseased spikes in RGB images captured by UAV devices. GSEYOLOX-S represents an improvement over the YOLOX-S model by integrating the SimAM attention module and GhostConv, which were trained and tested on spike datasets annotated with disease severity levels (Mao et al. 2023). Separately, Yang et al. (2024) modified the lightweight YOLOv8 model using GhostConv, Focal CIoU loss, and a C-faster module, proving its viability for mobile deployment. However, current image-based methods for FHB detection and quantification primarily focus on spikes and lack specialized spikelet-level algorithms for automated DSR measurement. Major challenges include the absence of spikelet-annotated datasets for FHB, difficulty in detecting diseased spikelets as small objects, and insufficient spatial encoding of densely arranged spikelets.

Existing datasets for FHB detection in wheat can be categorized into two types based on imaging modalities: hyperspectral image datasets (Almoujahed et al. 2022; Mustafa et al. 2024; Rangarajan et al. 2022) and RGB image datasets (Bao et al. 2025; Rößle et al. 2023; Zhou et al. 2024). While hyperspectral imaging-based methods achieve higher detection accuracy, large model parameter sizes and complex feature engineering hinder their deployment on resource-constrained mobile devices. Conversely, RGB image annotations primarily focus on spike-level labeling and lack fine-grained spikelet-level annotations (Mao et al. 2023; Zhang et al. 2023a). These datasets fail to capture and analyze disease information at individual spikelet granularity, which limits the development of deep learning models for automated DSR measurement. Therefore, constructing a spikelet-level annotated dataset is critical for establishing benchmark data and advancing the development of fine-grained disease detection algorithms.

Existing object detection approaches for diseased spikelets achieve precise FHB detection but face three key limitations: poor small-object feature extraction, high parameter complexity, and difficulty in mobile phenotyping deployment (Bao et al. 2025; Shoaib et al. 2023). Current small-object detection models are primarily built on generic object detection frameworks (Li et al. 2022b; Peng and Wang 2022). These models utilize multi-scale feature fusion through serial structures, simple feature addition, and fixed-scale convolutions but lack adaptive scale variation capture (Chandana and Ramachandra 2022; Sun et al. 2024). These limitations result in excessive computational costs while risking semantic information loss for diseased spikelets during deep network propagation (Chen et al. 2023b; Luo et al. 2022). Zhao et al. (2023) proposed BiTNet, a lightweight object detection network combining Transformer and a bidirectional Feature Pyramid Network (FPN). BiTNet enhances multi-scale feature learning through an efficient convolutional aggregation mechanism, effectively enriching both deep and shallow feature representations. Tao et al. (2020) proposed hierarchical multi-scale attention based on adjacent-scale combination learning and a Region-of-Interest-guided dynamic attention mechanism to distinguish and enhance scale-specific features. Although iterative prediction-based supervision in Transformer detectors enables dynamic hyperparameter tuning and multi-scale feature extraction, these processes drastically escalate parameter counts (Zhang et al. 2023b). Furthermore, detecting diseased spikelets as small targets is still challenging, especially with large-scale changes and complex backgrounds, which often cause false detections or missed cases (Chen et al. 2023b; Van Quyen and Kim 2023). Thus, dynamic multi-scale feature fusion methods are needed to efficiently capture scale variations while reducing computational overhead and strengthening feature representation (Li et al. 2024).

Another key challenge in diseased spikelet detection is dense occlusion, where overlapping spikelets blur boundaries and obscure contextual information, hindering accurate target localization (Kong et al. 2024; Wang et al. 2021). Current deep convolutional networks primarily rely on attention mechanisms to enhance local positional features (Ma et al. 2024; Ullah et al. 2024; Zeng et al. 2024). Bao et al. (2024) proposed a parallel channel–spatial attention module to detect FHB targets in complex backgrounds. Attention mechanisms enhance multi-scale feature representation by integrating spatial and channel information. However, their inherent design involves dimensional expansion, which fails to balance model complexity (De Santana Correia and Colombini 2022). Specifically, concatenating features across scales drastically increases channel dimensions, resulting in excessive computational overhead. By contrast, scale-aware methods offer a novel way to resolve spatial relationships among densely packed spikelets (Chen et al. 2021; Lin et al. 2023). These methods emphasize dynamic understanding of scale variations, focusing on handling scale distribution shifts and aligning features across different scales. By adopting a dynamic learning paradigm to efficiently capture scale changes, these models improve sensitivity to the positional variations of spikelets while reducing computational complexity. For instance, Chen et al. (2021) applied scale-aware techniques within a FPN to address domain adaptation issues caused by variations in lighting. The authors designed an image-level adaptive mechanism in the Faster R-CNN framework, training separate domain classifiers for each scale-specific feature; however, this approach significantly increased model complexity. Lin et al. (2023) leveraged serial mechanisms in Transformer models, progressively expanding receptive fields through iterative convolutions. Though capturing local dependencies in shallow layers and global dependencies through feature aggregation in deeper layers, it struggles to refine scale differences among targets within the same feature map and remains computationally intensive. For diseased spikelet detection in complex field scenarios, such methods still lack flexibility in multi-scale feature fusion.

To address the challenges of the lack of spikelet-level annotated datasets, the difficulty in detecting small diseased spikelet targets, and dense spikelet occlusion, in this study, we constructed a spikelet-level annotated image dataset for FHB and designed an automated DSR measurement framework. The framework consists of the lightweight detection network FHBDSR-Net for diseased and healthy spikelet detection. Automated DSR calculation is then performed through spikelet counting based on the detection results. FHBDSR-Net integrates a Multi-scale Feature Enhancement (MFE) module to improve diseased spikelet representation, the Inner-EfficiCIoU loss function to mitigate localization errors, and a Scale-Aware Attention (SAA) mechanism to enhance spatial encoding and inter-spikelet correlations. This framework is designed for deployment on resource-constrained devices, enabling portable DSR phenotyping. By establishing a spikelet-level annotated dataset and providing a benchmark for fine-grained disease detection, this approach advances precision phenotyping research, with implications for wheat resistance breeding and agricultural sustainability.

## Results

### Annotation information for disease detection in wheat spikelets

**Fig. 2:**
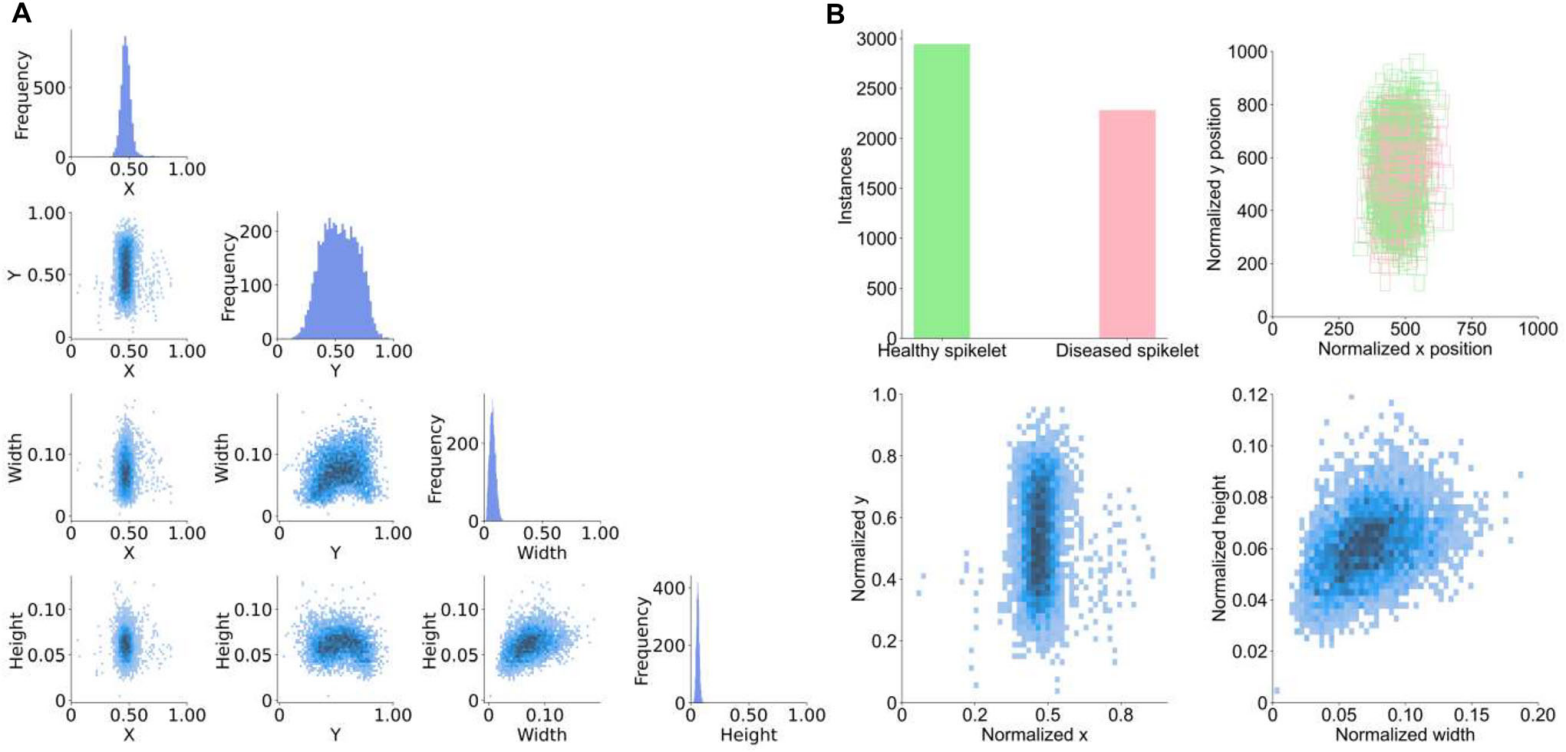
Statistical analysis of spikelet-level annotations. **A** Distribution of normalized bounding box dimensions (width and height) and centroid coordinates (x and y). **B** Spatial distribution and category statistics of diseased and healthy spikelets

We conducted a comprehensive analysis of the annotation information used for disease detection in wheat spikelets, focusing on three key aspects: the size distribution of bounding boxes, the spatial position distribution, and the correlation between parameters. The results are summarized in Table 1. We evaluated the normalized width and height of annotated bounding boxes to quantify the scale characteristics of the dataset. Given the limitations of the traditional absolute-scale definition due to differences in image sizes in non-MSCOCO datasets, we adopted the relative-scale criterion: when both the normalized width and height ratios of the bounding boxes are less than 0.1, the targets are defined as small objects (Chen et al. 2016). The scales of the bounding boxes of both diseased and healthy spikelets met this standard (Table 1), confirming that spikelets are typical small targets, providing empirical support for the architectural design of the MFE module. Further observation of size distribution showed that the widths are mainly concentrated in the range of 0–0.15, and the heights are concentrated in the range of 0–0.1 (Fig. 2A), again verifying that small targets dominate the dataset.

**Table 1:** Statistical analysis of small targets in dataset annotations

Scale ratio	Mean	Variance	Standard deviation
Bounding box width of diseased spikelet	0.0764	0.0006	0.0240
Bounding box height of diseased spikelet	0.0645	0.0002	0.0134
Bounding box width ratio of healthy spikelet	0.0705	0.0008	0.0278
Bounding box height ratio of healthy spikelet	0.0592	0.0002	0.0129

Note: The scale ratio refers to the proportion of the length and width of the annotation box to the length and width of the original image, respectively

We analyzed the normalized centroid coordinates of the spikelet bounding boxes to help reveal the spatial layout patterns within the spike. The positions of the bounding boxes on the X-axis are concentrated in the 0.4–0.6 range and follow a normal distribution, while on the Y-axis, the bounding boxes are dispersedly distributed in the range of 0.3–0.7, with a peak bias toward 0.5. This distribution pattern is closely related to the vertical and elongated morphology of wheat spikes. Overall, the annotations exhibit a central clustering tendency, but there are some outliers, likely due to deviations in camera angle or changes in distance during imaging. In addition, diseased spikelets are mainly distributed in the middle regions of wheat spikes. Correlation analysis of the horizontal and vertical position variables revealed a vertical clustering pattern in the bounding box annotations. This finding provides a basis for integrating spatial context encoding into the SAA module, which enhances the detection robustness in dense target scenarios.

Analysis of the correlation between the width and height of the spikelet bounding boxes revealed a significant positive correlation, that is, larger targets tend to have both greater widths and heights, while smaller targets maintain smaller but coordinated sizes, reflecting the proportional scaling characteristics of target dimensions. However, the scattered distribution of the dimensional ratios also indicates differences in the aspect ratios of individual spikelets (Fig. 2B), reflecting the diversity of target shapes. This proportional scaling rule inspired the design of the regression loss function, prompting us to introduce a scale-adjusted auxiliary box mechanism and guiding the design of the Inner-EfficiCIoU loss function to optimize the accuracy of target localization. Correlation analysis also revealed no significant linear relationship between positional parameters (x, y) and dimensional parameters (width, height).

### Performance evaluation of diseased spikelet detection

We conducted comprehensive experiments to compare the performances of the proposed FHBDSR-Net with advanced object detectors in terms of detection accuracy and lightweight characteristics (Table 2). FHBDSR-Net achieved an average precision of 89.2% for detecting healthy spikelets (AP_h_) and 93.8% for detecting diseased spikelets (AP_d_), outperforming state-of-the-art detectors. This improvement benefits from the multi-scale feature enhancement framework and scale-aware attention module, which strengthen the spatial representation of small spikelets and enhance the average detection precision for diseased spikelets. Compared with the baseline model GELAN-C, the parameters were reduced by 71.7% for FHBDSR-Net and by 69.7% for FLOPs. Notably, compared to YOLOv7, FHBDSR-Net achieved 80% parameter reduction and 70% computational load reduction while mean average precision (mAP) was improved by over 6%, demonstrating high-precision performance in resource-constrained environments. Although GELAN-C achieved the highest inference speed through its aggregated architecture and structural re-parameterization, FHBDSR-Net maintained a comparable model size and complexity to other lightweight detectors like YOLOv5-S and GELAN-S while achieving a better balance between accuracy and scale for disease detection. Compared with ultra-lightweight models such as YOLOv10-Nano and YOLOv11-Nano, FHBDSR-Net showed superior detection accuracy with only a slight increase in parameters within a controllable range while maintaining inference speed advantages. These results verify that FHBDSR-Net preserves lightweight characteristics while delivering high performance in detection tasks, demonstrating significant deployment potential for automated agricultural disease detection and intelligent applications.

**Table 2:** A comprehensive performance comparison of FHBDSR-Net and mainstream object detectors

Model	P↑ (%)	R↑ (%)	AP_h_↑ (%)	AP_d_↑ (%)	mAP↑ (%)	F1↑ (%)	Params↓ (M)	FLOPs↓ (G)	FPS↑
SSD-MobileNetV2 (Liu et al. 2016)	85.9	54.3	70.9	74.0	72.5	66.5	3.7	**1.4**	–
YOLOv3 (Redmon and Farhadi 2018)	85.9	81.5	87.1	91.2	89.1	83.6	103.7	283.0	32
YOLOv4-Tiny (Bochkovskiy et al. 2020)	82.0	82.6	84.6	82.3	83.5	82.0	5.9	6.8	–
YOLOv5-S (Jocher et al. 2020)	87.4	81.5	84.5	91.2	87.9	84.3	7.0	15.8	92
YOLOv6-Nano (Li et al. 2022a)	85.1	79.3	84.5	89.0	86.8	82.1	4.2	11.8	145
YOLOv7 (Wang et al. 2023a)	81.4	75.6	82.8	87.4	85.1	78.4	36.5	103.2	135
YOLOv8-S-Ghost (Sohan et al. 2024)	85.0	81.9	88.5	86.9	87.7	83.4	5.9	16.1	62
YOLOv8-S (Sohan et al. 2024)	82.4	82.7	86.2	90.0	88.1	82.5	11.1	28.6	93
RT-DETR-L (Zhao et al. 2024)	88.0	**87.1**	**91.2**	88.4	89.8	**87.5**	32.0	103.4	29
YOLOv9-S (Wang et al. 2024c)	80.8	85.2	88.2	91.4	89.8	82.9	8.2	33.8	46
GELAN-S (Wang et al. 2024c)	81.3	84.7	87.1	91.3	89.2	83.0	5.9	22.6	55
GELAN-C (Wang et al. 2024c)	**88.4**	86.2	88.3	91.2	89.8	87.3	25.4	102.5	**192**
YOLOv10-Nano (Wang et al. 2024a, b)	83.0	83.3	84.5	91.3	87.9	83.1	**2.7**	8.4	49
YOLOv10-S (Wang et al. 2024a, b)	84.3	83.3	85.1	91.8	88.5	83.8	8.1	24.8	42
YOLOv11-Nano (Khanam and Hussain 2024)	85.9	81.4	88.2	91.6	89.9	83.6	2.6	6.3	43
FHBDSR-Net (this work)	85.8	86.1	89.2	**93.8**	**91.5**	85.9	7.2	31.1	50

Note: *P* represents precision, *R* represents recall, AP_h_ represents the average precision of healthy spikelet detection, AP_d_ represents the average precision of diseased spikelet detection, mAP represents the mean average precision, params represents the algorithm parameters, and FLOPs represents floating-point operations per second. FPS stands for frames per second. An upward arrow indicates that the higher the value, the better the performance, while a downward arrow indicates that the lower the value, the better the performance. The best results are in bold

**Fig. 3:**
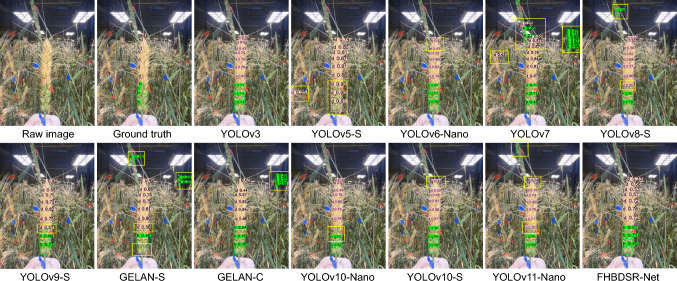
Visual comparison of inference results between FHBDSR-Net and mainstream object detectors. Yellow bounding boxes indicate missed, false positive, or overlapping detections. “h” and “d” denote healthy and diseased spikelets, respectively

We also conducted a comparative analysis of prediction effectiveness (Fig. 3). We selected test samples containing complex backgrounds, such as wheat leaves, stalk interference, and overlapping spikelets, to evaluate the detectors’ robustness and applicability in complex agricultural scenarios. Except for YOLOv3 and FHBDSR-Net, other detectors exhibited varying degrees of missed or false detections. YOLOv6-Nano showed overlapping detections when identifying diseased spikelets. Despite its lightweight design, its weak feature extraction capability limits the accuracy of DSR measurement. YOLOv3 produced relatively accurate results, but its low frame rate and high complexity render it less feasible in resource-constrained environments. GELAN-S excelled in terms of lightweight design and inference speed, but it still struggled with missed and false detections in complex backgrounds, a limitation also observed in YOLOv11-Nano. By contrast, FHBDSR-Net demonstrated superior robustness, effectively mitigating overlapping and missed detections while precisely capturing spikelet details, particularly for diseased spikelets.

### Ablation experiments on the MFE module in FHBDSR-Net

We evaluated the performance impact of the MFE Module across different network architectures (YOLOv7, YOLOv8-S, GELAN-S, and GELAN-C) through ablation experiments (Table 3). By comparing performance metrics before and after integrating the MFE Module, we validated its effectiveness in enhancing diseased spikelet detection tasks. Incorporating the MFE module into YOLOv7 increased Recall from 75.6% to 80.5% and improved AP_d_ from 87.4% to 89.7%, enhancing the performance of diseased spikelet detection. The lightweight YOLOv8-S also exhibited moderate improvements, with AP_h_ increasing from 86.2% to 88.0% and mAP rising from 88.1% to 89.9%. Notably, the performance gains from MFE were particularly pronounced for GELAN-C and GELAN-S: GELAN-C achieved an improvement from 91.2% to 92.7% for AP_d_ and from 89.8% to 91.8% for mAP, while YOLOv8-S showed an increase in F1-score from 82.5% to 85.3%, and GELAN-S showed an increase in F1-score from 83.0% to 84.9%. Although MFE introduces additional computational overhead, the increased complexity remains manageable in lightweight detectors, ultimately delivering substantial improvements in accuracy. Cross-architecture generalization experiments confirmed that the MFE Module effectively enhances detection performance while maintaining a favorable balance between precision and recall.

**Table 3:** Ablation study on the performance of the MFE module

Model	MFE	P↑ (%)	R↑ (%)	AP_h_↑ (%)	AP_d_↑ (%)	mAP↑ (%)	F1↑ (%)	Params↓ (M)	FLOPs↓ (G)
YOLOv7 (Wang et al. 2023a)	×	**81.4**	75.6	**82.8**	87.4	**85.1**	78.4	**36.5**	**103.2**
	✓	78.6	**80.5**	80.5	**89.7**	**85.1**	**79.5**	54.5	211.0
YOLOv8-S (Sohan et al. 2024)	×	82.4	82.7	86.2	90.0	88.1	82.5	**11.1**	**28.6**
	✓	**87.0**	**83.7**	**88.0**	**91.8**	**89.9**	**85.3**	11.9	33.8
GELAN-S (Wang et al. 2024c)	×	81.3	**84.7**	87.1	91.3	89.2	83.0	**5.9**	**22.4**
	✓	**85.7**	84.2	**88.0**	**92.0**	**90.0**	**84.9**	6.9	27.9
GELAN-C (Wang et al. 2024c)	×	**88.4**	**86.2**	88.3	91.2	89.8	**87.3**	**25.4**	**102.5**
	✓	86.5	84.7	**90.9**	**92.7**	**91.8**	85.5	28.4	116.2

The best results are in bold

**Fig. 4:**
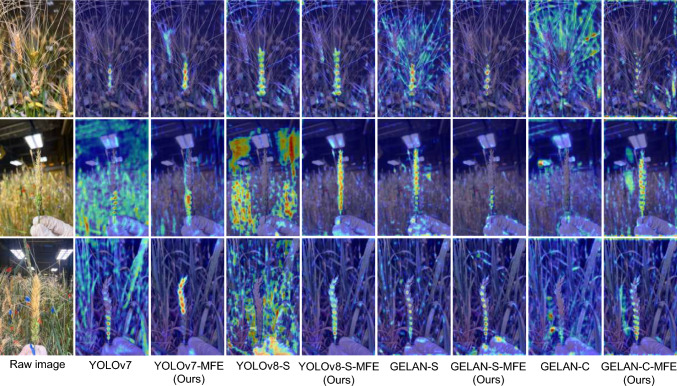
Comparison of feature visualization with and without the MFE module. Grad-CAM heatmaps highlight discriminative regions for spikelet detection in YOLOv7, YOLOv8-S, GELAN-S, and GELAN-C

To compare the feature visualization capacities of the detectors, we generated feature maps from small-object detection layers across morphologically diverse wheat varieties under varying scenarios (Fig. 4). We selected wheat spike samples with different levels of infection and morphological traits from the test set and compared changes in key feature regions, focusing on networks before and after integrating the MFE Module. Through quantitative and qualitative analysis of the visualization results, our findings validate the effectiveness of the MFE Module in enhancing feature representation for small targets, specifically diseased and healthy spikelets. Without the MFE Module, both the YOLOv7 and YOLOv8-S models exhibited heightened sensitivity to noise in complex backgrounds. This sensitivity led to high-intensity activation values around spikelets, adversely affecting the accuracy of object detection. By contrast, without the MFE Module, the GELAN-S and GELAN-C models tended to focus on fine-grained background details, such as awns, which may distract from critical target features. However, in complex environments, this excessive attention to background details can degrade the model’s decision-making accuracy. When the MFE Module was incorporated, all models demonstrated enhanced fusion capabilities for global spikelet features. The YOLOv7-MFE model achieved moderate improvements in suppressing background noise, enabling it to prioritize the overall morphological characteristics of wheat spikes. The YOLOv8-S-MFE model exhibited higher precision in detecting individual spikelets, effectively mitigating the impact of background interference on detection outcomes. The GELAN-S-MFE and GELAN-C-MFE models achieved superior attention allocation between healthy and diseased spikelets, significantly boosting detection accuracy. These feature visualization results demonstrate that the MFE Module improves detection accuracy for small-scale targets by strengthening multi-scale feature fusion capabilities while effectively alleviating interference in complex backgrounds.

### Ablation experiments on the SAA module in FHBDSR-Net

We integrated mainstream attention mechanisms into the GELAN-S model to evaluate the performance of the SAA module using a lightweight network (Table 4). SAA exhibited significant advantages, achieving a mAP of 91.1%, with a 1.9% improvement over the baseline due to its effective integration of spatial and scale enhanced features, enhancing performance in complex backgrounds. The convolutional block attention module (CBAM) (Woo et al. 2018), which combines spatial and channel information, focused more on disease features but needed to better detect healthy spikelets, likely due to the limited use of global information. Partial self-attention (PSA) (Wang et al. 2024a, b) showed strengths in recall and precision but struggled with intricate spikelets in complex backgrounds, leading to lower mAP and F1 scores than SAA and CBAM. SimAM, while efficient and parameter-free, did not provide improvements as notable as those of SAA. Despite increasing model complexity, the additional resource requirements for SAA remained manageable, offering strong feature enhancement capabilities that are well suited for high-accuracy detection under resource constraints.

**Table 4:** Ablation experiment comparing the performance of the SAA module with mainstream attention modules

Attention module	P↑ (%)	R↑ (%)	AP_h_↑ (%)	AP_d_↑ (%)	mAP↑ (%)	F1↑ (%)	Params↓ (M)	FLOPs↓ (G)
–	81.3	**84.7**	87.1	91.3	89.2	83.0	**5.87**	**22.4**
CBAM (Woo et al. 2018)	**86.5**	83.6	86.8	**94.4**	90.6	85.0	5.88	**22.4**
SimAM (Yang et al. 2021)	86.4	83.8	87.6	93.2	90.4	**85.1**	**5.87**	**22.4**
PSA (Wang et al. 2024a, b)	84.3	84.5	87.5	93.3	90.4	84.4	6.12	22.6
SAA (this work)	84.6	83.6	**89.0**	93.2	**91.1**	84.1	6.76	23.3

The best results are in bold

We also conducted parameter ablation studies on the SAA module within FHBDSR-Net, focusing on the impact of varying receptive field expansion scales through comparative performance evaluations (Table 5). The objectives were to explore optimized parameter combinations and investigate how different parameters influence the detection results of diseased spikelets. The scale-aware receptive field expansion is implemented via three parallel dilated convolutional layers. Notably, dilated convolutions preserve feature map resolution while altering receptive field scales, rendering parameter counts and computational complexity irrelevant in this experiment.

**Table 5:** Performance comparison of parameter ablation experiments using the SAA module

Dilation rate	P↑ (%)	R↑ (%)	AP_h_↑ (%)	AP_d_↑ (%)	mAP↑ (%)	F1↑ (%)	FPS↑
(1,3,5)	**85.8**	**83.8**	86.4	**93.4**	**89.9**	**84.8**	101
(1,4,9)	85.2	83.2	**87.6**	90.8	89.2	84.2	111
(1,6,12)	85.5	83.0	85.9	91.9	88.9	84.2	88
(1,12,18)	85.7	82.2	85.9	92.1	89.0	83.9	129
(3,5,7)	82.5	82.9	86.3	91.3	88.8	82.7	125
(6,12,18)	**85.8**	83.1	86.4	93.1	89.8	84.4	**133**

Note: The dilation rate represents the sizes of the three convolution kernels of the parallel dilated convolution layer in the SAA module. The best results are in bold

Retaining standard convolution (dilation rate = 1) during the SAA receptive field expansion phase significantly enhanced module performance. Specifically, the dilation rate combination (1, 3, 5) outperformed the (3, 5, 7) configuration by achieving a 3.3% improvement in precision, along with the highest mAP (89.9%) and F1-score (84.8%) across all classes. Furthermore, the combination (1, 4, 9) attained the highest average precision (87.6%) for healthy spikelets. However, excessively large dilation rates (e.g., 6, 12, 18) incurred a computational trade-off, reducing inference speed to 88 FPS. When standard convolution was preserved, other dilation rate variations had minimal impacts on overall mAP, with fluctuations confined within 1%. Conversely, excluding standard convolution-derived receptive fields allowed high-dilation combinations (6, 12, 18) to surpass low-dilation groups (3, 5, 7) by 1% mAP and 3.3% precision. These findings suggest that integrating standard convolution with high-dilation configurations enhances the differential perception of large-scale receptive fields, thereby improving performance. Moreover, prioritizing high-dilation combinations for large-scale feature extraction was particularly advantageous when standard convolution was omitted.

### Performance evaluation of diseased spikelet rate measurement

**Fig. 5:**
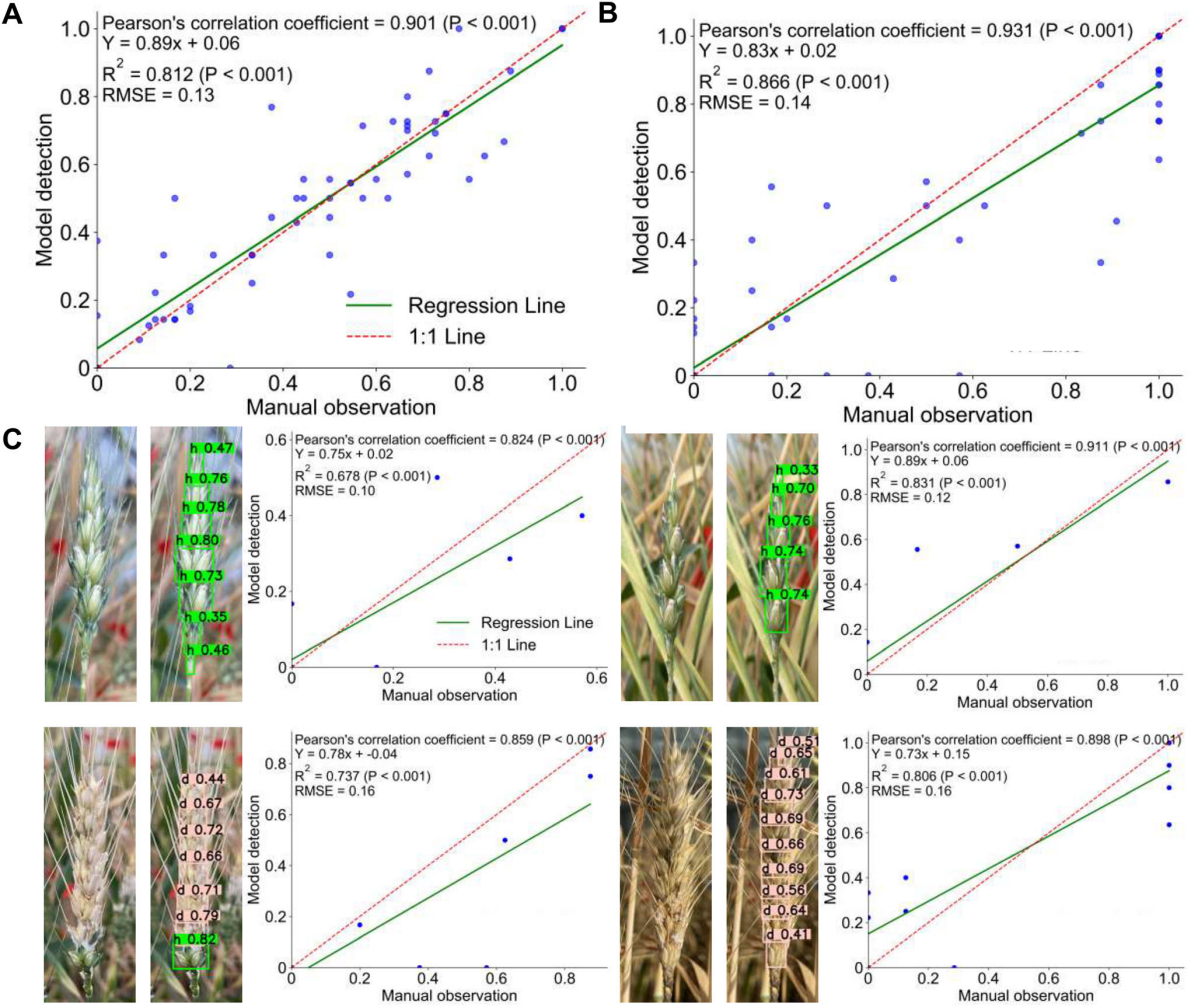
Correlation analysis between predictions of the DSR measurement algorithm and manual observation: **A** test set results. **B** Cross-cultivar validation. **C** Visual examples of detection instances with different cultivars. “h” and “d” denote healthy and diseased spikelets, respectively

We evaluated the predictive performance of FHBDSR-Net for measuring DSR on test set images (Fig. 5A). We compared the differences between the DSR predicted by FHBDSR-Net and manually observed values. In Fig. 5A, the horizontal axis represents the manually measured DSR, while the vertical axis represents the predicted values from FHBDSR-Net. The Pearson correlation coefficient between the detection results was 0.901 (*P* < 0.001), indicating a strong positive correlation. The regression equation Y = 0.89x + 0.06 suggests that FHBDSR-Net slightly underestimated the DSR, while the determination coefficient (*R*^2^) of 0.812 confirms the consistency of the model’s predictions. With a root mean square error (*RMSE*) of 0.13, the results demonstrate high reliability, indicating that the model can be effectively deployed in practical agricultural scenarios with complex conditions.

To further validate the model’s generalization ability and robustness, we collected an additional image set of 80 extreme samples comprising various wheat varieties and compared the results of FHBDSR-Net with manual observations (Fig. 5B). The Pearson correlation coefficient between the detection results was 0.931 (*P* < 0.001). The coefficient of determination (*R*^2^ = 0.866, *P* < 0.001) confirms excellent model fitting, while *RMSE* = 0.14 suggests minimal algorithmic error during phenotypic evaluation. Figure 5C presents the prediction results separately for different typical samples with various infection states and cultivars. For awned wheat varieties, phenotypic extraction achieved a Pearson correlation of 0.824 and *R*^*2*^ of 0.678, demonstrating the model’s effectiveness in capturing healthy spikelet characteristics. In awn-less wheat varieties, the Pearson correlation coefficient reached 0.911, indicating superior performance for cultivars with consistent textural features. The algorithm exhibited robust cross-cultivar generalization capability, particularly for wheat spikes with diverse morphological traits, including late-stage infected samples.

## Discussion

This study presents a lightweight framework for spikelet-level detection of FHB and automated measurement of DSR from RGB images of wheat spikes. We constructed a high-quality spikelet-level FHB dataset and designed FHBDSR-Net, an innovative lightweight detection network. Based on the detection results from FHBDSR-Net, the framework automatically calculates DSR and performs an efficient measurement process. This lightweight design is essential for portable phenotypic measurement in mobile scenarios.

Ensuring model compactness and measurement accuracy is crucial for practical deployment in farms and timely breeding decisions (Ali et al. 2025). Existing lightweight FHB detection methods include the GSEYOLOX-s model (Mao et al. 2023), which uses ghost convolution to achieve 99.23% classification accuracy with 8.06 M parameters and an inference speed of 47 FPS. A YOLOv4-based spike-level model (Hong et al. 2022) achieves 93.69% accuracy but requires a larger 56 M parameter size. Here, we designed a notably lighter solution for fine-grained spikelet-level detection. FHBDSR-Net achieves 91.5% detection accuracy with only 7.2 M parameters, making it 11% smaller than GSEYOLOX-s and 87% smaller than the YOLOv4-based model. FHBDSR-Net also offers a faster inference speed of 50 FPS. Our proposed model features an ultra-compact size and high-speed performance, making it highly suitable for mobile deployment, real-time field recognition, and use in agricultural environments with limited resources. Compared to previous methods, FHBDSR-Net better balances efficiency and accuracy, providing an ideal solution for edge computing and on-site applications.

Although our model can quickly and accurately extract DSR information, it still has certain limitations. First, when performing lightweight and high-throughput spikelet-level FHB detection, especially image-based field phenotyping, balancing accuracy and efficiency remains a core challenge. Most existing high-throughput detection studies used two-stage methods to achieve high accuracy (Liu et al. 2024; Wang et al. 2023c; Zhang et al. 2023a). However, we aimed to balance the accuracy of fine-grained DSR detection with model simplicity to achieve lightweight deployment. This balance may cause lightweight models to compromise on accuracy to improve efficiency (Shi et al. 2024). For example, insufficient feature extraction of dense small targets and interference from awns, such as overlapping spikelets and similar textures between spikelets and awns, may affect detection results (Liu et al. 2024). Second, factors such as light, vegetation background, weather conditions, and variety differences can affect model performance during field data collection (Shafik et al. 2023). Our dataset mainly comes from greenhouse environments, but it includes complex backgrounds and various lighting conditions to enhance model robustness. However, field samples have more complex diversity in terms of pathogen infection and disease symptoms (Al Masri et al. 2017), which poses challenges to data collection and the generalization ability of the model. In addition, our model relies on single-image inference for DSR measurement, which has inherent perspective limitations. To address this issue, we enhanced the diversity of the training data. For example, we collected images of both the front- and backsides of spikelets to handle differences in light and perspective. This step reduced potential biases to some extent, improved the generalization ability of the model for single-view inputs, and lowered the risk of missed or false detections caused by data biases.

The lightweight detection model FHBDSR-Net extends spikelet-level DSR measurement in greenhouse scenarios and shows potential for mobile deployment. This model can facilitate high-throughput, fine-grained phenotyping and provide reliable references for breeding decisions. Future research should explore more efficient learning paradigms to reduce dependence on data quality, reduce costs, and overcome limitations in feature representation (Nazki et al. 2020). It is also necessary to expand the dataset to include more field conditions and address the influence of uncontrolled factors such as lighting and weather. Finally, a multi-view, multi-dimensional FHB detection model could be developed to extract spatially related phenotypes with higher precision.

## Materials and methods

### Dataset preparation

The dataset used in this study consists of RGB images of FHB-infected wheat collected at the National Key Laboratory of Crop Genetic Improvement greenhouse facility of Huazhong Agricultural University, China (30.48° N, 114.36° E). The wheat growth environment, including temperature, relative humidity, and lighting conditions, was maintained under controlled parameters. FHB inoculum was uniformly sprayed onto healthy wheat spikes using an atomizer. The inoculated spikelets were covered with transparent plastic bags for 12 h to ensure sufficient pathogen infection.

Image acquisition commenced 21 days post-inoculation, when diseased spikelets exhibited conspicuous symptoms during the flowering stage (D’Angelo et al. 2014; Freije and Wise 2015), facilitating subsequent data annotation and model training. To ensure data diversity, imaging accounted for variations in growth stages and disease resistance across different wheat cultivars. Photographs were taken with the camera parallel to wheat spikes at a distance of 15–25 cm. Each spike was imaged from both frontal and dorsal (180° rotated) perspectives to comprehensively capture disease characteristics (Table 6). Initial quality control involved eliminating blurred images caused by focusing failures or interference from greenhouse growth lights.

**Table 6:** Camera settings used for image acquisition

Parameter	Value
Device	iPhone 13
ISO sensitivity	40
Focal length	26 mm
Aperture	f/1.6

Annotation was performed using LabelImg (v1.8.6), with individual spikelets manually labeled as “diseased spikelet” or “healthy spikelet”. The labeling process, guided and supervised by FHB pathology experts, strictly followed domain-specific knowledge (Mahlein et al. 2019; Mesterhazy 2020) to ensure accurate identification and localization. Annotation files were stored in VOC dataset format (XML) for model training and evaluation. Visual inspection of annotations enabled expert verification of both category labels and bounding box positions. Initial annotations were revised based on expert feedback, followed by secondary validation before finalizing the annotation files.

The final dataset comprises 620 high-resolution RGB images with a resolution of 3,024 × 4,032 pixels in JPEG format. These images are split into training, validation, and test sets at an 8:1:1 ratio. A total of 5,222 bounding boxes were annotated, consisting of 2,282 for diseased spikelets and 2,940 for healthy spikelets. To address class imbalance and enhance model generalization, online data augmentation strategies were applied during training, including Scale, MixUp, Mosaic, and Copy & Paste augmentation. For each training batch, the probabilities of applying these methods were set to 0.9, 0.15, 1.0, and 0.3, respectively. These configurations follow empirical findings commonly adopted in mainstream object detectors (Wang et al. 2024c).

### Model design for DSR measurement

**Fig. 6:**
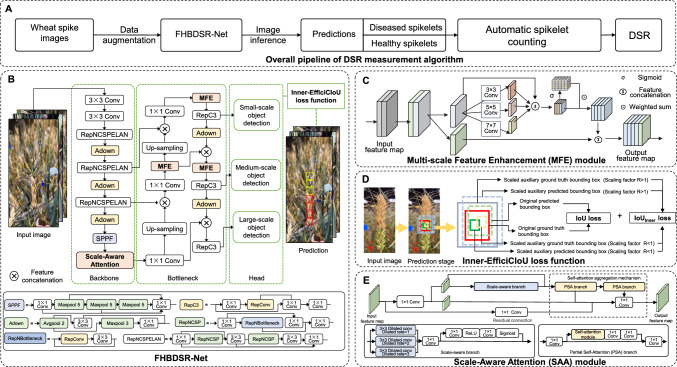
Design of the DSR measurement framework.** A** Overall pipeline of the DSR measurement algorithm. **B** General structure of the diseased spikelet detection network FHBDSR-Net.** C** Design of the multi-scale feature enhancement (MFE) module. **D** Visual explanation of the Inner-EfficiCIoU loss function. **E** Structure of the scale-aware attention (SAA) module

The DSR measurement algorithm is a systematic image-based intelligent analysis pipeline designed for accurate quantification of the DSR based on diseased spikelet detection. The algorithm consists of three stages (Fig. 6A). In the first stage, model training is performed. RGB images of individual wheat spikes are divided into patches. These patches are then fed into the FHBDSR-Net model after online data augmentation. The trained model outputs detection results that distinguish between diseased and healthy spikelets. Next, an automated spikelet counting module separately counts the number of diseased and healthy spikelets. Finally, the DSR value is calculated as the ratio of diseased spikelets to the total number of spikelets in each image. This algorithm provides an efficient, automated solution for assessing FHB infection status and extracting phenotype at the spikelet level.

FHBDSR-Net (Fig. 6B) is a multi-scale feature-enhanced detection network that processes 640 × 640 pixel images. Using two-class annotations (diseased and healthy spikelets), FHBDSR-Net generates corresponding bounding box predictions during inference. The network architecture integrates three key components: the novel MFE module, SAA module, and Inner-EfficiCIoU loss function. Key enhancements focus on backbone and bottleneck optimization, supplemented by the lightweight RepC3 module for parameter reduction (Zhao et al. 2024).

The backbone extracts low-level features through two 3 × 3 convolutional layers with batch normalization and SiLU activation, followed by Spatial Pyramid Pooling-Fast (SPPF) at its terminal to enhance spatial feature representation. A 1 × 1 convolutional layer then consolidates the extracted features in the bottleneck for efficient fusion in subsequent stages. To maintain a lightweight design and efficient gradient flow, the network employs the RepNCSPELAN module and the Asymmetric Downsampling (Adown) module. RepNCSPELAN integrates GELAN’s architecture, utilizing RepNCSP and 3 × 3 convolutions as fundamental blocks to optimize feature extraction while preserving computational efficiency through sequential aggregation. Notably, the proposed SAA module captures multi-scale contextual information across different pathological regions of spikelets, while a partial self-attention mechanism encodes spatial disease distribution patterns, significantly enhancing feature representation for dense spikelet detection.

In the bottleneck, features are first compressed via 1 × 1 convolution before being up-sampled through nearest-neighbor interpolation and concatenated with the corresponding hierarchical features. The FPN structure is optimized by incorporating the MFE module, which combines with the RepC3 and Adown modules to fuse lightweight properties with multi-scale feature enhancement. This hybrid architecture effectively handles both small and medium-scale targets, making it particularly suitable for diseased spikelet detection while still being deployable on mobile devices. The detection head utilizes anchor boxes and stride-based processing across multi-scale feature maps to predict bounding box coordinates and class probabilities. The proposed Inner-EfficiCIoU loss function further refines localization accuracy through scale-adaptive auxiliary bounding box optimization.

### Design of the MFE module in FHBDSR-Net

The MFE module strengthens the spatial feature representation of smaller spikelets in RGB images by enhancing inner-layer multi-scale features, allowing for better distinction from complex backgrounds. Inspired by Chen and coworkers (Chen et al. 2023a), the MFE module balances computation with feature fusion simplification by extracting features from partial channels while enhancing the interaction between different scale feature information (Fig. 6C). During feature extraction, the generated feature maps across different channels share high similarity, resulting in substantial redundancy, which consumes computational resources and hampers efficient feature extraction. By randomly selecting specific channels for conventional operations while leaving the rest unprocessed, the partial convolution design reduces redundant feature computations. This lowers memory access, significantly reducing floating-point operations (FLOPs). Equations (1) and (2) illustrate the principle of dynamic feature-weighted enhancement:

1 $$W_{i} = \sigma \left( {Conv_{1 \times 1} \left( {Concat\left( {Conv_{{k_{i} }} \left( {X_{{C_{P} }} } \right)} \right)} \right)} \right),{ }X_{{C_{P} }} \in {\mathbb{R}}^{B \times C \times H \times W}$$

2 $$Y = Concat\left( {\left( {\mathop \sum \limits_{i = 1}^{n} W_{i} \odot \left( {Conv_{{k_{i} }} \left( {X_{{C_{p} }} } \right)} \right)} \right),X_{{C_{u} }} } \right),{ }Y,{ }X_{{C_{u} }} ,{ }X_{{C_{P} }} \in {\mathbb{R}}^{B \times C \times H \times W}$$

where ${\mathbb{R}}^{B\times C\times H\times W}$ represents a feature tensor with a batch size, channel count, feature map height, and width of *B, C, H*, and *W*, respectively. ${W}_{i}$ represents the weight maps of features at different scales, ${k}_{i}$ represents convolution kernels of various sizes, Y is the output feature of the module, ${X}_{{C}_{P}}$ represents the feature map processed through convolution, and ${X}_{{C}_{u}}$ represents the unprocessed original feature map. $\sigma$ denotes a sigmoid operation that determines the weights of features at different scales. $\odot$ denotes an element-wise multiplication operation.

The MFE module first sets a channel ratio for the input features, randomly splitting the channels into a portion with ${C}_{p}$ channels for subsequent processing and another portion with ${C}_{u}$ channels of unprocessed original feature maps. The sum of ${C}_{p}$ and ${C}_{u}$ equals the total number of input channels *C*. The module dynamically adjusts the weights of features at different levels through a sigmoid function and then performs a weighted fusion to produce dynamically enhanced output features. To maintain a lightweight design while handling complex features and further enhancing multi-scale feature representation, the design incorporates two convolutional layers with 1 × 1 kernel, which introduces residual connections to form an inverse residual block, increasing the channel capacity at intermediate layers to boost feature extraction capabilities.

### Design of the Inner-EfficiCIoU loss function in FHBDSR-Net

Inner-EfficiCIoU is a novel IoU that combines Inner-IoU (Zhang et al. 2023c) and EfficiCIoU (Ji et al. 2024). Inner-IoU introduces a scaling factor and auxiliary bounding boxes to accelerate the regression process, addressing the slow convergence of original IoU calculations, as described in Eqs. (3) and (4):

3 $$B_{l} = x_{c} - \frac{{w{*}R}}{2},{ }B_{r} = x_{c} + \frac{{w{*}R}}{2}$$

4 $$B_{t} = y_{c} - \frac{{h{*}R}}{2},{ }B_{b} = y_{c} + \frac{{h{*}R}}{2}$$

where ${B}_{l}$, ${B}_{r}$, ${B}_{t}$, and ${B}_{b}$ represent the left, right, top, and bottom boundaries of the auxiliary anchor box, respectively, with the origin of the image at the top-left corner (0,0). The width and height of the original anchor box are denoted by w and h, with the scaling factor R controlling the size of the auxiliary anchor box, similarly applied to the ground truth box (Fig. 6D). The final IoU follows the traditional IoU computation. Introducing a scaling factor enhances the sensitivity of IoU between the anchor box and prediction box. EfficiCIoU loss is defined in Eq. (5), which further refines the influence of the aspect ratio by calculating the width and height differences between the anchor box and the ground truth box, thereby accelerating loss convergence:

5 $$L_{EfficiCIoU} = 1 - IoU + \frac{{\rho^{2} }}{{c^{2} }} + \frac{{w_{d} }}{{cw^{2} }} + \frac{{h_{d} }}{{ch^{2} }} + v\cdot\alpha$$

IoU represents the intersection over union, while ${w}_{d}$ and ${h}_{d}$ denote the squared differences in width and height between the predicted and target boxes, which are used to measure the size discrepancy. ${\rho }^{2}$ represents the Euclidean distance between the center points of the predicted and target boxes. ${c}^{2}$ is the squared length of the diagonal of the smallest enclosing box covering both the predicted and target boxes, which is used to normalize the center point distance, ensuring that the distance error is independent of the box scale. ${cw}^{2}$ and ${ch}^{2}$ are the squared width and height of the smallest enclosing rectangle, ensuring that scale discrepancy is independent of box size. The weight factor adjusts the value of *v*:

6 $$L_{Inner - EfficiCIoU} = L_{EfficiCIoU} + IoU - IoU_{Inner}$$

The final loss function is shown in Eq. (6). ${L}_{EfficiCIoU}$ combines Inner-IoU and EfficiCIoU to accelerate convergence while maintaining robustness in the bounding box regression task.

### Design of the SAA module in FHBDSR-Net

The SAA module, inspired by Dilateformer (Jiao et al. 2023) and YOLOv10 (Wang et al. 2024a, b), splits the input feature map into two parts along the channel dimension using a 1 × 1 convolution layer (Fig. 6E). This enhances the ability of the model to detect spikelets of varying sizes. One part enters the scale sensing enhancement branch, while the other retains the original feature information. The scale-aware branch expands receptive fields at different scales by applying dilated convolutions with varying dilation rates in parallel. A perception weight map is generated through the perception information extraction block, and the feature map is applied to the input feature map of this branch. The process of extracting scale-aware information is illustrated in Eq. (7):

7 $$\tilde{X} = Concat_{{d \in \left\{ {1,2,3} \right\}}} \left( {W_{{d^{*} dc}} X + b_{d} } \right)$$

where $X$ represents the input feature map, $\widetilde{X}$ denotes the resulting multi-scale perception distribution map, ${W}_{{d}^{*}dc}$ refers to the dilated convolution operation with a dilation rate of $d$, and ${b}_{d}$ represents the bias vector corresponding to the convolution operation with dilation rate $d$. The dilation rates $d\in \left\{\text{1,2},3\right\}$ indicate the various dilation rates, with $d=1$ being the standard convolution block. The receptive field is progressively expanded through the feature fusion of dilated convolution blocks with increasing dilation factors, followed by a 1 × 1 convolution block to align the channels of the input feature map and two subsequent 1 × 1 convolution blocks with ReLU activation to generate the perception weight map, capturing salient feature information learned from different scales of receptive fields.

The scale sensing-enhanced features are then passed through the partial self-attention (PSA) module from YOLOv10 to enhance spatial information. The input feature map is split into two parts along the channel dimension using a 1 × 1 convolution block. The weighted features from each head are concatenated along the channel dimension. A 3 × 3 grouped convolution layer is then applied, where the number of groups equals the number of channels, performing independent convolution for each channel to obtain positional encoding. The result is mapped back to the input feature channels using a 1 × 1 convolution block. Considering the information loss of small-scale targets such as spikelets in forward propagation due to information bottlenecks, a self-attention layer aggregation strategy is employed, aggregating the output of each PSA block with the retained original features using a generalized efficient layer aggregation structure and integrating with 1 × 1 convolution to improve gradient flow efficiency. Specifically, the SAA module adopts a 1 × 1 convolution residual connection (He et al. 2016) between input and output features to perform projection mapping, further optimizing the gradient flow.

### Evaluation metrics

FHBDSR-Net employs evaluation metrics including precision, recall, AP, mAP, and F1. In addition, the FPS and FLOPs metrics are used to evaluate the inference speed and complexity of the model, respectively. Precision (P) indicates the proportion of instances correctly predicted as diseased or healthy spikelets. Recall (R) represents the proportion of actual spikelets the model correctly detects, indicating that the model can detect most diseased spikelets with few false negatives. Average precision (AP) represents the area of the PR curve of each class. mAP, one of the most widely used evaluation metrics (Everingham et al. 2010), refers to the sum of APs across each class used to evaluate the performance of object detection algorithms. F1 calculates the harmonic mean of P and R, comprehensively evaluating model performance for detecting diseased and healthy spikelets. Frames per second (FPS) indicate the number of image frames the spikelet object detection algorithm can process per second. Params (Parameters) and FLOPs indicate the training weight parameter count and the floating-point operations of each layer, respectively, which assess the model complexity.

The statistical indicators used to compare model prediction results with manual counting results include *R*^2^ and root mean square error (*RMSE*) (Willmott and Matsuura 2005), as shown in Eqs. (8) and (9):

8 $$R^{2} = 1 - \frac{{\mathop \sum \nolimits_{i = 1}^{n} \left( {x_{i} - y_{i} } \right)^{2} }}{{\mathop \sum \nolimits_{i = 1}^{n} \left( {x_{i} - \overline{x}_{i} } \right)^{2} }}$$

9 $$RMSE = \sqrt {\frac{1}{n}\mathop \sum \limits_{i = 1}^{n} \left( {x_{i} - y_{i} } \right)^{2} }$$

where *n* represents the number of test samples, ${x}_{i}$ represents the manually detected disease severity, ${y}_{i}$ represents the automatically calculated disease severity, and ${\overline{x}}_{i}$ represents the mean of ${x}_{i}$. The ${R}^{2}$ value measures the degree of fit between the predicted values and the manually recorded values. A high ${R}^{2}$ indicates that the model can accurately predict the diseased spikelet rate, reflecting the model’s superiority in quantitatively assessing disease severity. Finally, *RMSE* quantifies the difference between predicted and observed values, with lower values indicating more accurate model predictions and minor deviations.

## Data Availability

The dataset and code generated in this study are available at https://github.com/WeizhenLiuBioinform/Wheat-FHB-DSR-Measurement.
