# Correction: FHBDSR-Net: automated measurement of diseased spikelet rate of Fusarium Head Blight on wheat spikes

**DOI:** 10.1007/s42994-025-00250-3

**Published:** 2025-11-05

**Authors:** Ze Wu, Haowei Zhao, Zeyu Chen, Yongqiang Suo, Seena Joseph, Xiaohui Yuan, Caixia Lan, Weizhen Liu

**Affiliations:** 1 School of Computer Science and Artificial Intelligence, Wuhan University of Technology, Wuhan, 430070 China; 2 Hubei Hongshan Laboratory, College of Plant Science and Technology, Huazhong Agricultural University, Wuhan, 430070 China; 3 School of Applied Computing, Wales Institute of Science and Arts, UWTSD, Swansea, SA1 8EW UK; 4 Yazhouwan National Laboratory, Sanya, 572025 China; 5 Engineering Research Centre of Chinese Ministry of Education for Edible and Medicinal Fungi, Jilin Agricultural University, Changchun, 130118 China; 6 Sanya Science and Education Innovation Park of Wuhan University of Technology, Sanya, 572025 China

**Correction: aBIOTECH** 10.1007/s42994-025-00245-0

In this article the author “Yongqing Suo” should read “Yongqiang Suo”.

The original article has been corrected.

